# Structural Basis for Specific Recognition of Substrates by Sapovirus Protease

**DOI:** 10.3389/fmicb.2012.00312

**Published:** 2012-09-05

**Authors:** Masaru Yokoyama, Tomoichiro Oka, Hirotatsu Kojima, Tetsuo Nagano, Takayoshi Okabe, Kazuhiko Katayama, Takaji Wakita, Tadahito Kanda, Hironori Sato

**Affiliations:** ^1^Pathogen Genomics Center, National Institute of Infectious DiseasesTokyo, Japan; ^2^Department of Virology II, National Institute of Infectious DiseasesTokyo, Japan; ^3^Food Animal Health Research Program, Ohio Agricultural Research and Development Center, Department of Veterinary Preventive Medicine, The Ohio State UniversityWooster, OH, USA; ^4^Open Innovation Center for Drug Discovery, The University of TokyoJapan

**Keywords:** sapovirus protease, substrate recognition, P1 and P4 amino acid residues, 3-D models, amino acid diversity, mutagenesis, 3-D pharmacophore, inhibitor screening

## Abstract

Sapovirus (SaV) protease catalyzes cleavage of the peptide bonds at six sites of a viral polyprotein for the viral replication and maturation. However, the mechanisms by which the protease recognizes the distinct sequences of the six cleavage sites remain poorly understood. Here we examined this issue by computational and experimental approaches. A structural modeling and docking study disclosed two small clefts on the SaV protease cavity that allow the stable and functional binding of substrates to the catalytic cavity via aromatic stacking and electrostatic interactions. An information entropy study and a site-directed mutagenesis study consistently suggested variability of the two clefts under functional constraints. Using this information, we identified three chemical compounds that had structural and spatial features resembling those of the substrate amino acid residues bound to the two clefts and that exhibited an inhibitory effect on SaV protease *in vitro*. These results suggest that the two clefts provide structural base points to realize the functional binding of various substrates.

## Introduction

Sapovirus (SaV) is a non-enveloped RNA virus that belongs to the family Caliciviridae and causes gastroenteritis in humans and swine (Chiba et al., 1979, 2000; Guo et al., 1999; Hansman et al., 2007). The SaV genome is a single-stranded RNA that encodes two or three open reading frames (ORFs; Liu et al., 1995; Noel et al., 1997; Numata et al., 1997; Guo et al., 1999; Robinson et al., 2002). The ORF1 encodes six non-structural proteins (NS1, NS2, NS3, NS4, NS5, and NS6-7) and a structural protein, the capsid protein (VP1; Oka et al., 2006, 2009). The NS6-7 protein contains the chymotrypsin-like protease domain (the 3C-like protease; Oka et al., 2005a,b, 2007; Robel et al., 2008) and the RNA-dependent RNA polymerase domain (the 3-D-like polymerase; Fullerton et al., 2007; Bull et al., 2011). The ORF1 precursor protein is posttranslationally cleaved at six sites by the 3C-like protease (Oka et al., 2005b, 2006).

The SaV 3C-like protease domain comprises 146 amino acid residues (Oka et al., 2007). This enzyme cleaves the peptide bonds of specific dipeptides, such as the glutamic acid/glycine (E/G), glutamine/glycine (Q/G), and glutamic acid/alanine (E/A; Oka et al., 2006). However, these dipeptide motifs exist in the non-cleaved sites of the ORF1 polyprotein, indicating that additional amino acid residues are required for the specific recognition of substrates (Oka et al., 2006). In this regard, calicivirus proteases have a large cavity that can accommodate substrate peptides with several amino acid in lengths (Nakamura et al., 2005; Zeitler et al., 2006; Oka et al., 2007). It is conceivable that these substrate amino acid residues around the cleavage sites, termed the P4, P3, P2, P1, P1′, P2′, P3′, and P4′ sites, are all involved to some extent, either directly or indirectly, in the recognition and cleavage by protease. However, there must be a division of roles: previous studies on the calicivirus proteases consistently suggest more extensive involvement of the substrate amino acid residues upstream of the peptide bond of the cleavage sites in the cleavage by proteases (Wirblich et al., 1995; Sosnovtsev et al., 1998; Hardy et al., 2002; Belliot et al., 2003; Scheffler et al., 2007; Robel et al., 2008). In the case of SaV, the substrate P1 and P4 amino acid residues in particular are physicochemically more conserved among different SaV strains (Oka et al., 2009) and more sensitive to the substitutions (Robel et al., 2008; Oka et al., 2009). Therefore, these amino acid residues may provide the specific contact sites with SaV protease. However, due to the lack of structural information on SaV protease and its substrates, such interaction remains unclear.

Recent advances in the hardware and software for biomolecular simulation and bioinformatics have rapidly improved the precision and performance of these techniques. We have applied some of these techniques, in combination with experimental methods, to understand the structural and evolutionary basis of the virological phenomena (Oka et al., 2007, 2009; Motomura et al., 2008, 2010; Naganawa et al., 2008; Shirakawa et al., 2008; Yokoyama et al., 2010, 2012; Ode et al., 2011; Sakuragi et al., 2012). In this study, by combining methods of homology modeling, the automated ligand docking, Shannon entropy analysis, site-directed mutagenesis, and *in silico* screening of SaV inhibitors, we studied the structural basis for the substrate recognition by SaV protease.

## Materials and Methods

### Structural modeling of SaV protease docked to the substrate octapeptides

We first constructed a ligand-free protease domain model of the SaV Mc10 strain (Oka et al., 2005b; GenBank accession number: AY237420) by the homology modeling technique (Sanchez et al., 2000; Baker and Sali, 2001) as described previously (Oka et al., 2007). The modeling was performed using tools available in the Molecular Operating Environment (MOE; Chemical Computing Group, Inc., Montreal, QC, Canada). As the modeling template, we used the high-resolution crystal structure of norovirus 3C-like protease at a resolution of 1.50 Å [Protein Data Bank (PDB) code: 2FYQ; Zeitler et al., 2006] because, like SaV, the norovirus belongs to the family Caliciviridae, and thus the protease sequence shows a higher identity to the SaV protease sequences (about 25% identity) than to the other available 3C-like protease sequences of viruses. We applied the multiple sequence alignment approach (Baker and Sali, 2001) using the reported 3C-like proteases to minimize misalignments of the target and template sequences, as described previously (Oka et al., 2007; Shirakawa et al., 2008). The sequences used for the alignment included those of the rhinovirus 3C-like protease (PDB code: 1CQQ; Matthews et al., 1999), the poliovirus 3C-like protease (PDB code: 1L1N; Mosimann et al., 1997), and the hepatitis A virus 3C-like protease (PDB code: 1QA7; Bergmann et al., 1999). The alignment was done with the alignment tool MOE-Align, and homology modeling was done with the tool MOE-Homology in MOE. We optimized the 3-D model thermodynamically via energy minimization using the MOE and an AMBER99 force field (Ponder and Case, 2003). We further refined the physically unacceptable local structure of the models based on a Ramachandran plot evaluation using MOE. The 3-D models of the six octapeptides corresponding to the six cleavage sites of the ORF1 precursor protein of the SaV Mc10 strain (NS1/NS2, NS2/NS3, NS3/NS4, NS4/NS5, NS5/NS6-7, and NS6-7/VP1) were constructed using the Molecular Builder module in MOE. Subsequently, the thermodynamically and physically optimized protease models were used to construct protease-substrate complex models. Individual octapeptide models were docked with the optimized SaV protease domain model described above, using the automated ligand docking program ASEDock2005 (Goto et al., 2008) operated in MOE as described previously (Yokoyama et al., 2010). Default setting in ASEDock2005 was applied for the search of the candidate docking structures, and the structures with the best docking score expressed by the arbitrary docking energy (*U*_dock_) in ASEDock2005 (Kataoka and Goto, 2008) were selected for the analysis of the protease-substrate interaction sites.

### Analysis of amino acid diversity with information entropy

The amino acid diversity at individual sites of the SaV protease domain was analyzed with Shannon entropy scores as described previously (Sander and Schneider, 1991; Mirny and Shakhnovich, 1999; Oka et al., 2009). The amino acid sequences of the protease domain of various human SaV strains from different geographic regions in the world were obtained from GenBank (the number of sequences is 19; accession numbers: X86560, AY694184, AY237422, AY237423, AY646853, AY646854, AJ249939, AY237420, AY237419, AY646855, AY603425, AJ786349, DQ058829, DQ125333, AY646856, DQ125334, DQ366344, DQ366345, DQ366346). The amino acid diversity within the SaV protease population was calculated using Shannon’s formula (Shannon, 1948):

$$H\left(i\right)=-\sum_{x_{i}}p\left(x_{i}\right)\log_{2}p\left(x_{i}\right)\;\left(x_{i}=G,A,I,V,\ldots \ldots \right),$$

where *H(i)*, *p(x_i_)*, and *i* indicate the amino acid entropy (*H*) score of a given position, the probability of occurrence of a given amino acid at the position, and the number of the position, respectively. An *H* score of zero indicates absolute conservation, whereas 4.4 bits indicates complete randomness. The *H* scores were displayed on the 3-D structure of the SaV protease model constructed above.

We also calculated the Shannon entropy by considering the physicochemical properties of amino acid residues, i.e., the chemical properties and size of side chains as described previously (Oka et al., 2009). For analysis of the diversity in the chemical properties, the amino acid residues were classified into seven groups: acidic (D,E), basic (R,K,H), neutral hydrophilic (S, T, N, Q), aliphatic (G, A, V, I, L, M), aromatic (F, Y, W), thio-containing (C), and imine (P). For analysis of the diversity in the size of side chains, the amino acid residues were classified into four groups: small (G, A, C, S), medium-small (T, V, N, D, I, L, P, M), medium-large (Q, E, R, K), and large (H, F, Y, W). The *H* scores were plotted on the 3-D structure of the SaV protease model.

### Site-directed mutagenesis of the SaV protease domain

The detailed strategy of the mutagenesis for the SaV protease domain has been described previously (Oka et al., 2005b, 2006). Briefly, we used the full-length cDNA clone of the genome of the SaV strain Mc10 (pUC19/SaV Mc10 full-length; GenBank accession number: AY237420) as a starting material for the mutagenesis. We constructed nine SaV Mc10 full-length mutant cDNA clones. Site-directed mutagenesis was performed using a GeneTailor Site-Directed Mutagenesis System (Invitrogen). The oligonucleotides used for the site-directed mutagenesis were as follows (the codons corresponding to changed amino acid(s) are indicated in lowercase): for T1085A, 5′-GTGGTTGTCACAGTTgcaCACGTGGCCTCTGCG-3′; for Y1156A, 5′-ATCACGGTCCAGGGGgctCACCTGCGCATCATA-3′; for K1167A, 5′-ATGGATACCCAACAgcgCGTGGGGACTGTGGCAC-3′; for R1168A, 5′-GATACCCAACAAAGgctGGGGACTGTGGCACAC-3′; for K1167E, 5′-ATGGATACCCAACAgagCGTGGGGACTGTGGCAC-3′; for R1168E, 5′-ATGGATACCCAACAAAGgagGGGGACTGTGGCACAC-3′; for K1167AR1168A, 5′-ATGGATACCCAACAgcggcgGGGGACTGTGGCAC-3′; and for K1167ER1168E, 5′-ATGGATACCCAACAgaggagGGGGACTGTGGCAC-3′. The T1085AY1156A mutant was generated with the above Y1156A primer using methylated DNA of the T1085A as the template. All the mutant clones constructed were subjected to the sequencing of the entire genomic cDNA region to verify the absence of unnecessary mutations leading to amino acid changes.

### *In vitro* transcription-translation assay

*In vitro* transcription-translation with a rabbit reticulocyte system was performed using the TNT T7 Quick for PCR DNA kit (Promega, Madison, WI, USA) as described previously (Oka et al., 2005b). Briefly, a template for the *in vitro* transcription-translation, containing the entire ORF1, was prepared by PCR amplification using the full-length cDNA clone. The primers used for the amplification were as follows. The forward primer containing a T7 promoter sequence (underlined) and a translation initiation codon (bold) was 5′-GGATCCTAATACGACTCACTATAGGGAACAGCCACC**ATG** gcttccaagccattctacccaatagag-3′; and the antisense primer containing a stop codon (bold) was 5′-T_30_**TTA**-ttctaagaacctaacggcccgg. The PCR product (3 μl) was mixed with 20 μl of TNT T7 PCR Quick Master Mix (Promega) and 2 μl of Redivue Pro-mix L- [^35^S] *in vitro* cell-labeling mix (GE Healthcare Biosciences, Piscataway, NJ, USA). The mixture was incubated at 30°C for 3 or 16 h and subjected to SDS-polyacrylamide gel electrophoresis (SDS-PAGE). The translation products separated by electrophoresis were blotted onto a PVDF membrane (Immobilon-P; Millipore, Bedford, MA, USA) using a semi-dry electroblotting apparatus (ATTO; Tokyo). The radiolabeled proteins were detected by a BAS 2500 Bioimage Analyzer (Fuji Film, Tokyo).

### Immunoprecipitation

For the detection of NS1 (p11) and NS5 (VPg), which were undetectable with the above assay system, we performed immunoprecipitation before the SDS-PAGE as described previously (Oka et al., 2005b, 2006, 2009). Briefly, 10 μl of the *in vitro* transcription-translation reaction mixture was diluted with 80 μl of RIPA lysis buffer containing 50 mM Tris, pH 7.4, 150 mM NaCl, 0.25% deoxycholic acid, 1% NP40, and 1 mM EDTA (Upstate, Lake Placid, NY, USA) and incubated with 5 μg of anti-A (anti-NS1) or ant-D (anti-NS5) antibodies raised against *E. coli*-expressed recombinant proteins (aa 1–231 for NS1 and aa 941–1055 for NS5; Oka et al., 2005b). After incubation for 1 h on ice, 25 μl of a suspension of Protein A Magnetic Beads (New England Biolabs) and 900 μl of RIPA buffer were added. The mixture was gently rotated at 4°C for 1 h and then washed three times with 1 ml of RIPA lysis buffer. The immunoprecipitated proteins were resuspended in 20 μl of SDS-PAGE sample buffer and heated at 95°C for 5 min prior to analysis with 5 to 20% Tris-Gly polyacrylamide gel. The proteins were blotted onto an Immobilon-P polyvinylidene difluoride membrane (Millipore). Immunoprecipitated radioactive proteins were detected with a Bioimage Analyzer BAS 2500 (Fuji Film).

### The chemical compound library

Chemical compounds (139,369 compounds, molecular weights 42–2986) were obtained from the Open Innovation Center for Drug Discovery (The University of Tokyo, Tokyo, Japan). The compound library database of this center provides information on the molecular formula, molecular weight, hydrogen-bond donor-acceptor numbers, topological polar surface area (TPSA), and other physicochemical parameters of the compounds for pharmacophore-based *in silico* drug screening.

### Pharmacophore-based *in silico* screening

Pharmacophore-based *in silico* screening was done using tools available in the MOE. We created a pharmacophore query with a substrate feature using the Pharmacophore Query Editor tool in MOE. Pharmacophore-based *in silico* screening was done by the Pharmacophore Search module in the MOE using the created query.

### Drug susceptibility assay

The susceptibility of SaV protease to the synthetic small chemical compound was determined by means of an *in vitro* trans cleavage assay as follows. A radiolabeled full-length Mc10 ORF1 polyprotein containing a defective protease (Pro^mut^; Oka et al., 2005b) or a non-radiolabeled partial Mc10 ORF1 polyprotein (NS6-7-VP1) containing a functional protease (Pro^wt^; Oka et al., 2006) was separately expressed using the *in vitro* transcription/translation system (Oka et al., 2011). The PCR primer pairs used for the preparation of DNA template for the expression of the NS6-7-VP1 were as follows. The forward primer was 5′-GGATCCTAATACGACTCACTATAGGGAACAGCCACC**ATG**gctcccacaccaattgttac-3′, including the T7 promoter sequence (underlined) and a start codon (bold); and the antisense primer was 5′-T_30_**TTA**-ttctaagaacctaacggcccgg, including a stop codon (bold). Twenty microliter of the non-radiolabeled products containing Pro^wt^ was mixed with 1 μl of 2 mM inhibitor candidate in DMSO and incubated for 10 min at room temperature. Then 10 μl of the radiolabeled full-length ORF1 polyprotein (Pro^mut^) was added to the Pro^wt^-inhibitor mixture and incubated at 30°C for 20 h, and subjected to the SDS-PAGE analysis as described above. To quantitate the proteolytic activity of the SaV protease, we measured the intensity of the band corresponding to the NS4-NS5 intermediate processing product with Typhoon 7500 (GE Healthcare), due to the lack of overlapping non-specific products of the *in vitro* translation around the NS4-NS5. The chemical compound concentrations resulting in a 50% reduction of the NS4-NS5 intermediate protein production of the drug-free control were determined on the basis of the dose-response curve and defined as the IC_50_ values of the SaV proteolysis activity.

### Structural modeling of SaV protease docked to chemical compounds

Structural models of the chemical compounds were constructed using the Molecular Builder tool in MOE. Individual compounds were docked with the SaV protease domain model using the automated ligand docking program ASEDock2005 (Goto et al., 2008) operated in MOE as described above.

## Results

### Structural modeling of SaV proteases docked to the substrate octapeptides

**Figure 1:**
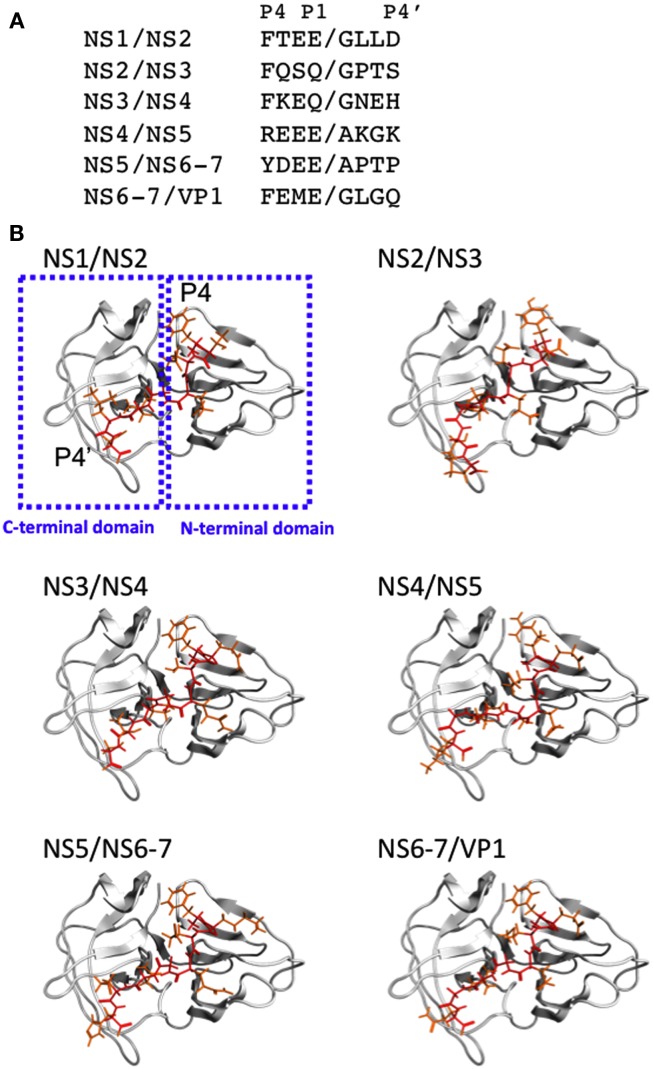
**Structural models of SaV protease docked to the substrate octapeptides**. **(A)** Sequences of the six cleavage sites of the SaV ORF1 polyprotein are shown with one-letter amino acid codes. Slashes represent the peptide bonds cleaved by the protease. **(B)** Structural models of the SaV protease-substrate complex. The 3-D structural model of the protease domain was constructed by homology modeling and thermodynamically and physically refined as described previously (Oka et al., 2007). The 3-D structural models of the octapeptides corresponding to the six authentic cleavage sites of the SaV Mc10 ORF1 were constructed by using the Molecular Builder tool in MOE. The optimized protease model was docked to individual octapeptides using the automated ligand docking program ASEDock2005 (Goto et al., 2008) operated in MOE as described previously (Yokoyama et al., 2010). Red and orange sticks indicate main and side chains of the octapeptides, respectively.

To obtain structural insights into the protease-substrate interactions at the atomic level, we constructed a 3-D model of the intact protease domain of the SaV Mc10 strain, which were docked to octapeptides corresponding to the six authentic cleavage sites (P4–P4′ sites) of the ORF1 polyprotein of the Mc10 strain (see Materials and Methods for details; Figure 1). The amino acid sequences of the six octapeptides are very different from each other (Figure 1A). Despite the variation, the octapeptides bound to the protease with the same orientation in the clefts of the protease (Figure 1B); the P1–P4 amino acid residues bound to the cleft between the N- and C-terminal domains, whereas the P1′–P4′ amino acid residues bound to the cleft on the C-terminal domain. The docking positions were functionally reasonable, because they allowed the cleavage sites of the octapeptides to be placed near the amino acids essential for the catalytic activity of the SaV protease, i.e., H^31^, E^52^, C^116^, and H^131^ (Oka et al., 2005b). Other docking positions caused docking results with very poor docking scores and did not fulfill the functional requirement for the catalytic reaction.

**Figure 2:**
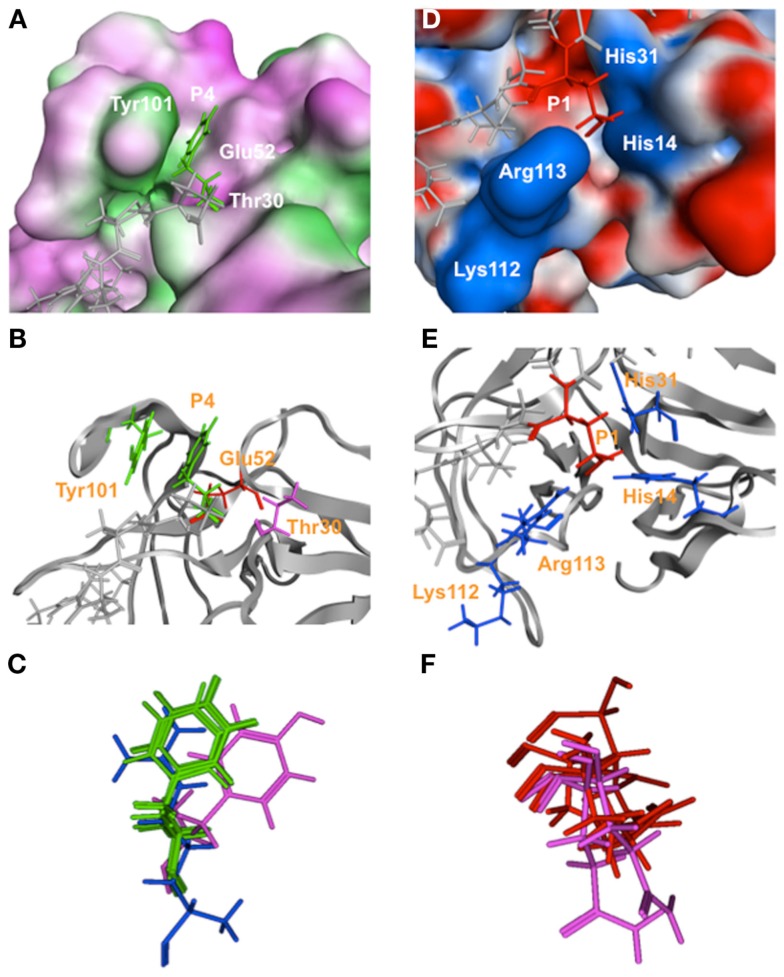
**Close-up views of the interaction sites in the protease-substrate complex models**. The sites of interactions between the SaV Mc10 protease and the side chains of octapeptides at the P4 **(A–C)** and P1 **(D–F)** sites are highlighted. Upper panels show the cleft formed by T^30^, E^52^, and Y^101^, and the positively charged cleft formed by the H^14^, H^31^, K^112^, and R^113^ of the proteases that are bound to a side chain of the NS6-7/VP1 octapeptide at the P4 **(A)** and P1 **(D)** sites. Middle panels show the relative configurations of side chains of the protease bound to a side chain of the NS6-7/VP1 octapeptide at the P4 **(B)** and P1 **(E)** sites. Bottom panels show the superposed structures of the side chains of the P4 **(C)** and P1 **(F)** amino acid residues of the 6 cleavage sites in the SaV protease-substrate complex models. **(C)** Green, magenta, and blue sticks represent the side chains of phenylalanine, tyrosine, and arginine, respectively, at the P4 site in the substrate-protease complexes. **(F)** Red and magenta sticks represent the side chains of glutamic acid and glutamine, respectively, at the P1 site in the substrate-protease complexes.

The protease-peptide complex models disclosed two interaction sites that were common to the six bound peptides. First, the substrate amino acid residues at the P4 position were exclusively placed in a thin cleft, termed cleft 1, that was formed by threonine (T), glutamic acid (E), and tyrosine (Y) at positions 30, 52, and 101 of the protease domain (T^30^, E^52^, and Y^101^, respectively; Figures 2A–C). An aromatic ring of the phenylalanine (F) or Y at P4 of the octapeptides of the NS1/NS2, NS2/NS3, NS3/NS4, NS5/NS6-7, and NS6-7/VP1 cleavage sites (Figure 1A) was positioned such that an aromatic stacking could be generated with the Y^101^ in the protease cleft 1 (Figures 2A–C). The steric configuration of the aromatic rings of the P4 amino acid residues in the bound state was very similar except for the Y of the NS5/NS6-7 cleavage site (Figure 2C). In the case of the NS4/NS5 peptide, the P4 amino acid is the arginine (R; Figure 1A) and was arranged near the side chain of the E^52^ (Figure 2C).

Second, the substrate amino acid residues at the P1 site were exclusively placed in a small positively charged cleft, termed cleft 2, that was formed by the histidine (H), H, lysine (K), and R at positions 14, 31, 112, and 113 of the protease domain (H^14^, H^31^, K^112^, and R^113^, respectively; Figures 2D–F). In four out of the six cleavage sequences the P1 amino acid is negatively charged (E; Figure 1A) that could interact electrostatically with the side chains of the positively charged cleft 2 of the protease (Figure 2D). In the case of the NS2/NS3 and NS3/NS4, the P1 amino acid was glutamine (Q; Figure 1A) which is hydrophilic and thus could cause electrostatic interactions via a polarized charge. The steric configuration of the side chains of the P1 amino acid residues at the bound state was very similar (Figure 2F). The simulated docking between the protease and the substrate having alanine substitutions at P1 and P4 positions resulted in a docking position similar to that for the wild-type substrate, whereas the docking score was reduced to about 1/2. Collectively, these results suggest that the interactions at the P1 and P4 sites of the substrates play a key role in the substrate recognition, as suggested in the previous experiments (Robel et al., 2008; Oka et al., 2009).

### Amino acid diversity of human SaV protease

**Figure 3:**
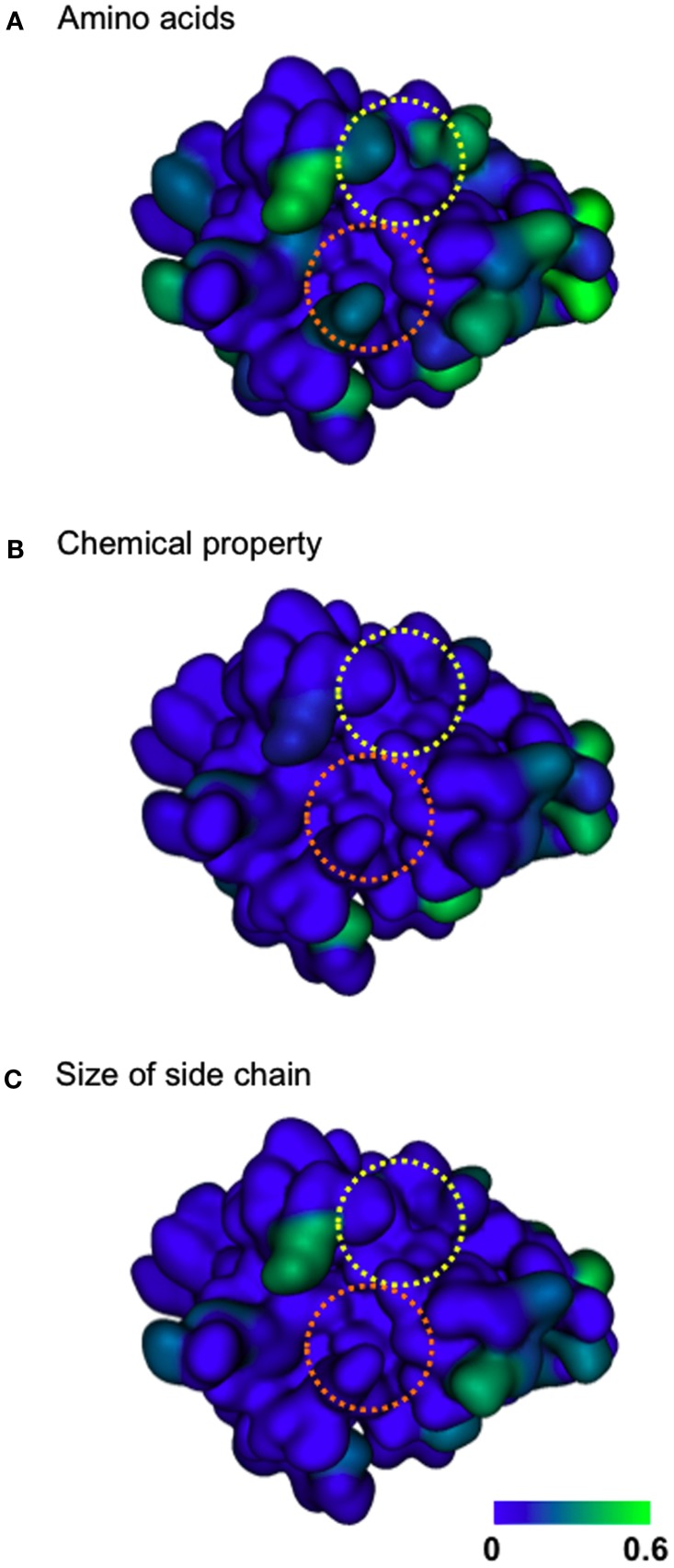
**Diversity of SaV protease amino acid residues**. The amino acid diversity at individual sites of the SaV protease domain was analyzed with information entropy as described previously (Oka et al., 2009). The Shannon entropy *H* was calculated with Shannon’s formula (Shannon, 1948) based on amino acid residues **(A)**, chemical properties **(B)**, and the size of the side chain **(C)** using amino acid sequences of the SaV full-length protease domain from GenBank (*N* = 19). For analysis of the diversity in the chemical properties, the amino acid residues were classified into seven groups: acidic (D,E), basic (R,K,H), neutral hydrophilic (S, T, N, Q), aliphatic (G, A, V, I, L, M), aromatic (F, Y, W), thio-containing (C), and imine (P). For analysis of the diversity in the size of the side chain, the amino acid residues were classified into 4 groups: small (G, A, C, S), medium-small (T, V, N, D, I, L, P, M), medium-large (Q, E, R, K), and large (H, F, Y, W). The *H* scores are plotted on the 3-D structure of the SaV protease model, where an *H* score of zero indicates absolute conservation. Yellow and orange dotted circles indicate clefts 1 and 2, respectively.

To obtain evolutionary insights into the protease-substrate interactions, we analyzed the amino acid diversity of the protease domain among various human SaV strains in the public database. Full-length human SaV protease domain sequences were collected from GenBank (*N* = 19) and used to calculate the Shannon entropy scores, *H* (Shannon, 1948), in order to analyze the diversity of individual amino acid residues in the SaV population as described previously (Oka et al., 2009). The *H* scores generally ranged from 0.0 to 0.6 bits (Figure 3A), indicating that the diversity of the SaV protease is relatively small, as seen in many viral enzymes. The variable sites were essentially located on the surface region, indicating that some exposed regions of the SaV protease allow amino acid changes (Figure 3A, greenish sites). Although less extensive, some variation was detected at Y^101^ and R^113^ in the clefts 1 and 2, respectively (Figure 3A, two dotted circles). However, when the *H* scores were calculated on the basis of chemical properties or the size of the amino acid residues, they were nearly zero throughout the substrate-binding cleft (Figures 3B,C). Similarly, the protease amino acid residues, which constitute a large cavity for the binding of entire octapeptides, were sometime variable but highly conserved in the context of the chemical properties and sizes of the side chains (Figures 1B and 3). Thus the SaV protease appears to restrict extensive changes in the shape and chemical properties of the substrate-binding surface for its survival.

### Site-directed mutagenesis of SaV protease

**Figure 4:**
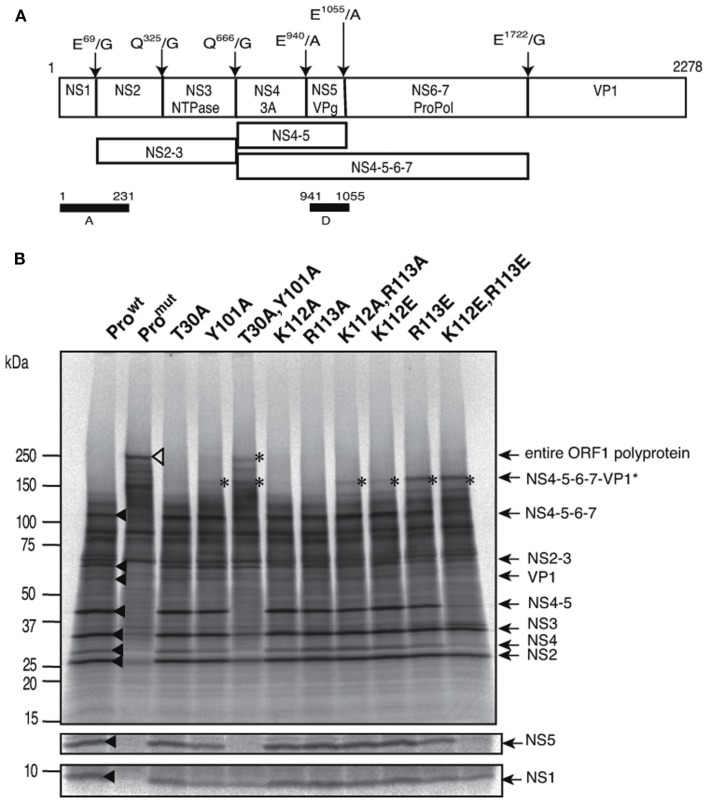
**Site-directed mutagenesis of the substrate interaction sites of SaV Mc10 protease**. **(A)** Proteolytic cleavage map of the SaV Mc10 ORF1 polyprotein and the processing intermediates (Oka et al., 2006). Black bars indicate the protein segments, A and D, used to raise polyclonal antibodies for detection of the NS1 and NS5 proteins, respectively. **(B)** SDS-PAGE of ^35^S-labeled *in vitro* translation products of SaV Mc10 ORF1 containing various protease mutants. NS1 and NS5 were detected by immunoprecipitation using anti-A or anti-D polyclonal antibodies as described previously (Oka et al., 2005b, 2006, 2009). Mc10 ORF1 containing functional protease (Pro^wt^) and a defective mutant lacking in the proteolysis activity (Pro^mut^) were included as described previously (Oka et al., 2005b). Newly appearing products when compared to Pro^wt^ are indicated by asterisks. Size markers are shown on the left. Mc10 ORF1-specific proteins (Oka et al., 2005b, 2006) are shown on the right.

Consistent with the above structural and diversity data, we previously reported that the E^52^ in cleft 1, as well as H^14^ and H^31^ in cleft 2, are essential to maintain proper processing by SaV protease (Oka et al., 2007). To obtain further insights into the biological roles of clefts 1 and 2 in the proteolysis of the SaV precursor polyprotein, we performed additional site-directed mutagenesis using a full-length clone of SaV Mc10 strain (Oka et al., 2005b). The Mc10 ORF1 encodes a polypeptide of 2278 amino acid residues, where the six cleavage sites have been experimentally determined (Oka et al., 2006; Figure 4A). A total of nine mutants of the SaV protease domain were constructed using the Mc10 ORF1. Full-length ORF1 precursor proteins having a single or double mutations in the protease domain were expressed using the *in vitro* transcription-translation system, and the processing products were analyzed by gel electrophoresis as described previously (Oka et al., 2005b, 2006, 2007, 2009). The Mc10 functional protease (Pro^wt^) and a defective mutant completely lacking the proteolysis activity (Pro^mut^; Oka et al., 2005b) were used as positive and negative controls of the proteolysis, respectively.

When the ORF1 containing the Pro^wt^ was expressed, nine products corresponded in size to the mature proteins NS1, NS2, NS3, NS4, NS5, and VP1, and relatively stable intermediate proteins, such as NS2-3, NS4-5, and NS4-5-6-7 were detected (Figure 4B, lane Pro^wt^, black arrowheads; Oka et al., 2005b, 2006, 2009). These products were undetectable in the Pro^mut^ ORF1 sample, and instead a product corresponding to the ORF1 polyprotein was detected (Figure 4B, lane Pro^mut^, open triangle; Oka et al., 2005b, 2006, 2009). A single alanine substitution at T^30^ in the cleft 1, K^112^ in the cleft 2, or R^113^ in the cleft 2 of viral protease resulted in a processing pattern similar to that of Pro^wt^ (Figure 4B, lanes T30A, K112A, and R113A). On the other hand, a single alanine substitution at Y^101^ in the cleft 1 (Y101A), a single acidic substitution at K^112^ or R^113^ in the cleft 2 (K112E and R113E), and double mutations in each cleft (T30AY101A and K112ER113E) resulted in abnormality of the precursor processing, i.e., an increase in accumulation of the full-length ORF1 polyprotein and/or the NS4-5-6-7-VP1 intermediate protein (Figure 4B, asterisks). In the samples expressing ORF1 with T30A/Y101A or K112E/R113E double mutations, processing products corresponding to the NS5 and NS4-5 disappeared almost completely (Figure 4B, lanes 5 and 11, respectively).

### Pharmacophore-based *in silico* screening for the lead compounds of SaV protease inhibitors

**Figure 5:**
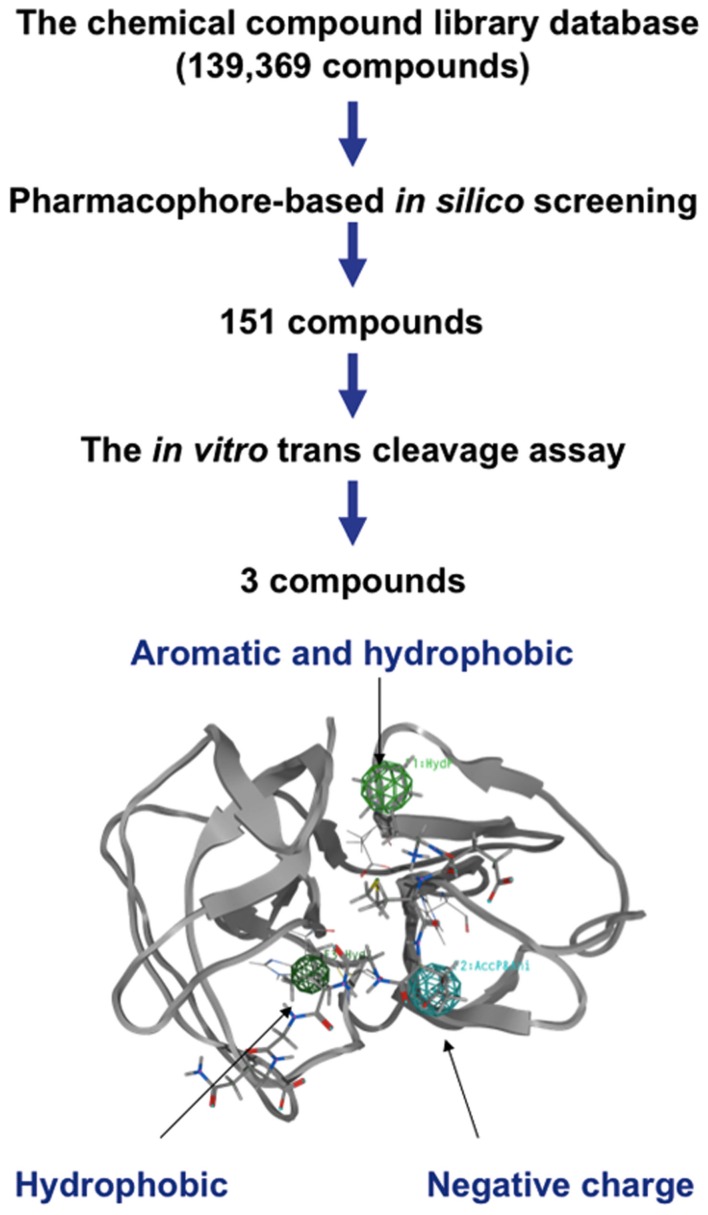
**Flow chart for screening chemical compounds against SaV protease**. Pharmacophore-based *in silico* screening (Schuster et al., 2006a,b; Kirchmair et al., 2007) was applied to extract the lead compounds having structural features that resembled those of the substrates of SaV protease. One hundred and fifty-one compounds exhibiting some similarities to the authentic substrates were further assessed with respect to their inhibitory activity against SaV protease. With this strategy, we could obtain three compounds that inhibited the processing of the SaV ORF1 polyprotein.

**Figure 6:**
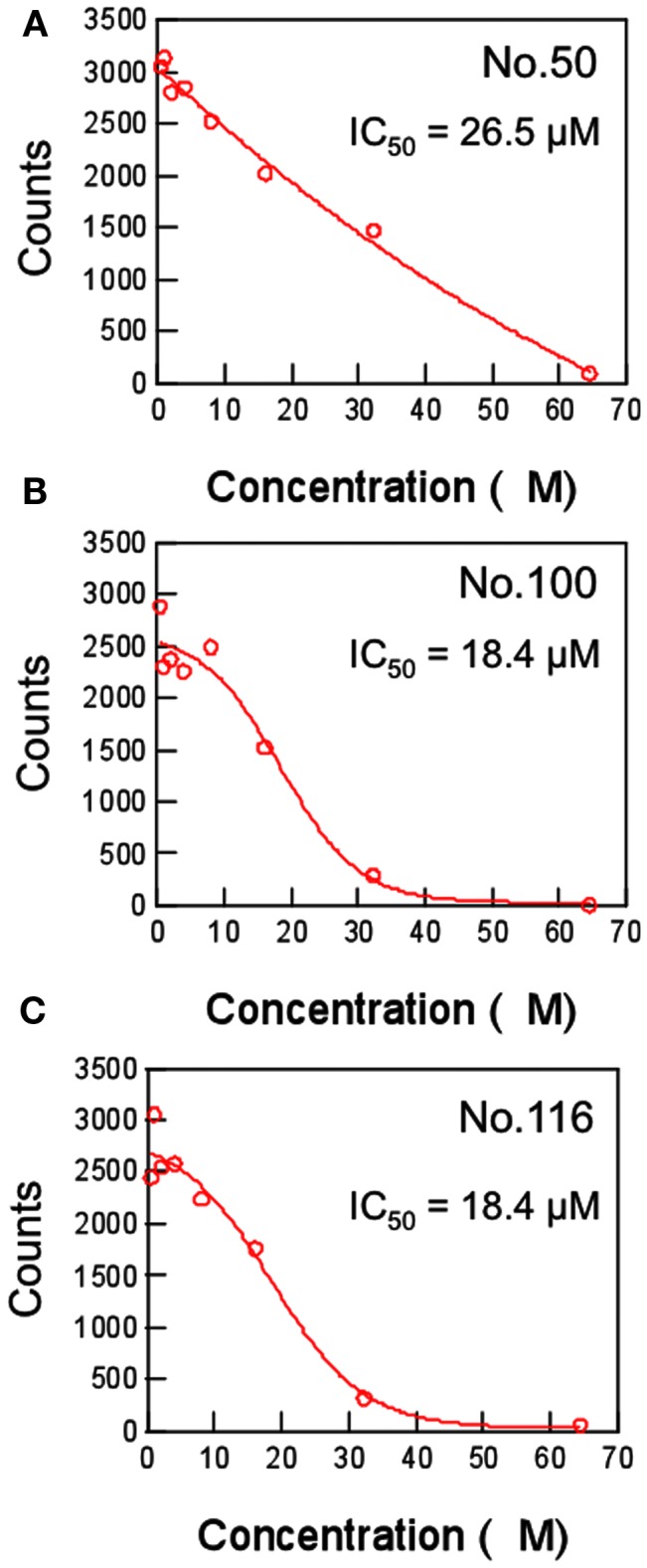
**Dose-response curves of the inhibitors against SaV protease**. The inhibitory effects of the three chemical compounds that were screened for their structural similarity to the authentic substrates of SaV protease were determined with an *in vitro* trans cleavage assay. A radiolabeled full-length Mc10 ORF1 polyprotein containing a defective protease (Pro^mut^; Oka et al., 2005b) or a non-radiolabeled partial Mc10 ORF1 polyprotein (NS6-7-VP1) containing a functional protease (Pro^wt^; Oka et al., 2006) was separately expressed using the *in vitro* transcription/translation system. The translation products were mixed and incubated in the presence of increasing concentrations of the indicated compounds at 30°C for 20 h. The intensity of the radioactive band corresponding to the NS4-NS5 product was measured with Typhoon 7500 and plotted in relation to the compound concentrations. **(A)** Compound No.50. **(B)** Compound No.100. **(C)** Compound No.116.

To further assess the role of the clefts 1 and 2 in the ligand binding, we performed a pharmacophore-based *in silico* screening of protease inhibitors. A total of 139,369 compounds (molecular weights 42–2986) were screened for the lead molecules that contain an aromatic-ring-like portion resembling the P4 amino acid, a negatively charged portion resembling the P1 amino acid, and a hydrophobic portion resembling the P1′ amino acid, being arranged at similar 3-D positions with the authentic substrates (Figure 5). The hydrophobic portion resembling the P1′ amino acid was included to better mimic the authentic substrate structures. A total of 151 lead compounds matched to the category were then subjected to the *in vitro* trans cleavage assay of the SaV Mc10 ORF1 polyprotein. With this screening, we could obtain three compounds that inhibited processing of the SaV ORF1 at IC_50_ values of 18.4–26.5 μM (Figure 6).

**Figure 7:**
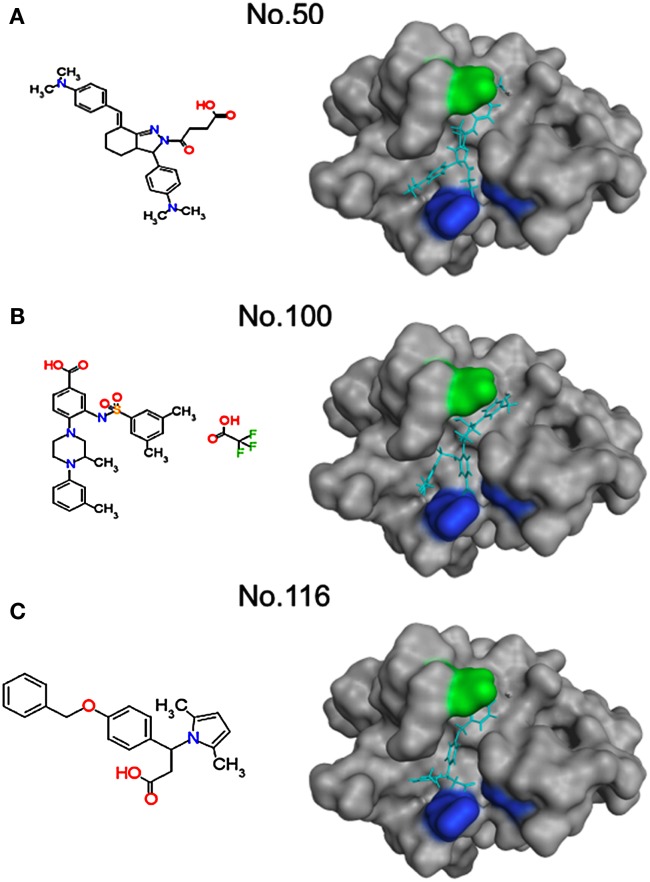
**Structural models of SaV protease docked to the inhibitors**. Molecular formulas of the inhibitors (left) and structural models of the inhibitor-protease complexes (right) are shown. Molecular models of the three chemical compounds having anti-SaV-protease activity were constructed using the Molecular Builder tool in MOE. Individual compounds were docked to the SaV protease domain model using the automated ligand docking program ASEDock2005 (Goto et al., 2008). Light blue sticks in the protease indicate inhibitors. Greenish and bluish portions of the protease indicate an aromatic and hydrophobic site and positively charged site, respectively. **(A)** Compound No.50. **(B)** Compound No.100. **(C)** Compound No.116.

We then analyzed how the lead compounds bound to the SaV Mc10 protease by docking simulation (Figure 7). As expected, these compounds were predicted to bind to the protease at the same interaction sites by which the authentic substrates bound to the protease. The aromatic-ring-like portion resembling the P4 amino acid bound to the thin cleft formed by T^30^, E^52^, and Y^101^ for the binding of the side chain of the P4 amino acid. The negatively charged portion resembling the P1 amino acid bound to the small positively charged pocket formed by the H^14^, H^31^, K^112^, and R^113^ for the binding of the side chain of the P1 amino acid.

## Discussion

The viral proteins that support viral replication and make up the viral particle are often translated as part of polyprotein precursors. Viral protease catalyzes cleavage of the precursor protein and thus plays an essential role in the viral life cycle. In this study, by combining computational and experimental approaches, we studied the structural basis for the substrate recognition by SaV protease. The results obtained in this study were consistent with each other and disclosed novel structural base points of the protease for the attractive interactions with specific structures of ligands.

Using a homology modeling and a docking tool, we first examined the physical interactions of SaV protease and octapeptides corresponding to the six authentic cleavage sites of the SaV ORF1 polyprotein. Despite the marked sequence variation of the octapeptides, they were bound to the protease in the same orientation in the structural models (Figure 1). The results suggested that there might be common interaction sites that served as fulcrums to direct the orientation of the octapeptides. Consistently, the models disclosed two interaction sites that were shared with the six peptides and support the stable and functional binding of substrates to the catalytic cavity; the variable side chains at the P4 and P1 sites of the peptides were consistently bound to the two small clefts, termed clefts 1 and 2, respectively (Figure 2). The former participated in aromatic stacking interactions, whereas the latter participated in electrostatic interactions. These results are consistent with the previous findings that the P4 and P1 amino acid residues of the substrates play key roles in efficient proteolysis by SaV protease (Robel et al., 2008; Oka et al., 2009) and predicted that these two clefts could play a key role in substrate recognition via interactions with the P4 and P1 amino acid residues of substrates.

This prediction was assessed by several analyses. If the clefts played essential roles in recognition of substrates, spontaneous mutations that alter profoundly the physicochemical properties of the clefts should be suppressed for viral survival. Consistently our Shannon entropy study using protease sequences of various SaV strains from the world shows that the amino acid residues forming the clefts 1 and 2 are variable but highly conserved in terms of the chemical properties or the sizes of side chains (Figure 3). The results indicate that these clefts tolerate mutations in nature but resist a range of mutations that markedly alter the chemical properties or the shapes of the cleft surface. The findings are consistent with the above structure-based prediction on the function of the clefts 1 and 2. These clefts are located on the surface of the large cavity of the protease. Therefore, the restrictions in the variation in two clefts are likely to be caused by functional constraints for the essential interactions.

Moreover, we examined whether a range of mutations that markedly alter the physicochemical properties of the clefts indeed could result in aberrant processing of the SaV precursor polyprotein. Our site-directed mutagenesis study showed that a single mutation in cleft 1 (T30A) or in cleft 2 (K112A or R113A) caused little detectable damage in the processing of the viral precursor polyprotein, showing a tolerance to mutations as indicated by our information entropy study. Notably, however, (i) a single mutation that causes a loss of aromatic stacking interaction (Y101A) in the cleft 1, (ii) a single mutation that causes a loss of the electrostatic interaction in the cleft 2 (K112E or R113E), and (iii) double mutations within the clefts unexceptionally resulted in incomplete processing (Figure 4). The results indicate that the abnormal processing was caused only by single mutations that could extensively alter the chemical properties of the clefts. The data agree with the entropy data and again suggest the acceptability of variation in the two clefts under functional constraints.

Finally, we performed *in silico* screening of SaV protease inhibitors on the basis of the above structural and biological information. The screening of the 139,369 compounds *in silico* led to the identification of the 151 compounds that resembled the structural and spatial features of the P4 and P1 amino acid residues of authentic substrates (Figure 5). From them, we could experimentally identify the three compounds that inhibited proteolysis of the SaV precursor polyprotein *in vitro* (Figure 6). As expected, these compounds were predicted to bind to the SaV protease at the two clefts via similar attractive interactions with the authentic ligands (Figure 7). These results provide additional evidence that two clefts on the SaV protease cavity play a key role in the ligand recognition by providing the structural base points for the specific attractive interactions.

Notably, six cleavage sites of SaV precursor polyprotein also differ with respect to their susceptibility to the SaV protease, with the NS2/NS3, NS4/NS5, and NS5/NS6-7 sites being consistently more resistant to the cleavage than the NS1/NS2, NS3/NS4, and NS6-7/VP1 sites (Oka et al., 2005b, 2006, 2009). In this regard, it is of note that the P4 position of the NS4/NS5 site of human SaV is exclusively arginine instead of an aromatic amino acid (Figure 1A) and that this arginine is conserved in all human SaV strains reported thus far (Oka et al., 2005b, 2006, 2009). This substitution at P4 position will abolish the aromatic stacking interaction in the cleft 1 and thus will attenuate attractive interactions between protease and the NS4/NS5 cleavage site. This possibility is well consistent with the experimental findings; the cleavage of the NS4/NS5 site is less efficient than that of the other sites (Oka et al., 2005b, 2006, 2009) and is more sensitive to the cleft 1 mutations than the other cleavage sites are (Figure 4, lane 5, NS5). Moreover, the attenuation of cleavage of the NS4/NS5 site was reversed simply by replacing the arginine with phenylalanine at the P4 site (Oka et al., 2009). These findings strongly suggest that the well-preserved arginine at the P4 position of the SaV NS4/NS5 cleavage site plays a key role in maintaining the distinct cleavability of precursor polyprotein by SaV protease.

In this study, we disclosed a novel 3-D pharmacophore containing two clefts on the cavity of the SaV protease, which can be used to identify the lead compounds of SaV protease inhibitors. SaV is one of the commonly detected pathogens in the acute gastroenteritis of both children and adults (Johansson et al., 2005; Harada et al., 2009; Iturriza-Gomara et al., 2009; Pang et al., 2009). Diarrhea is one of the greatest causes of mortality in children under age 5 in many countries (Boschi-Pinto et al., 2008), and the outbreaks of the acute gastroenteritis often seriously affects the clinical, economic, and social activities. Therefore, anti-viral compounds against SaV may be beneficial to some at-risk populations or communities. Thus far no anti-SaV inhibitors for the clinical use have been developed. Our findings will provide important clues to the unique specificity of the SaV protease, the regulation of SaV maturation, and the rationale design of anti-SaV inhibitors.

## Conflict of Interest Statement

The authors declare that the research was conducted in the absence of any commercial or financial relationships that could be construed as a potential conflict of interest.
